# Longevity of duodenal and peripheral T-cell and humoral responses to live-attenuated *Salmonella* Typhi strain Ty21a

**DOI:** 10.1016/j.vaccine.2018.05.114

**Published:** 2018-07-25

**Authors:** Shaun H. Pennington, Daniela M. Ferreira, Jesús Reiné, Tonney S. Nyirenda, Ameeka L. Thompson, Carole A. Hancock, Angela D. Wright, Stephen B. Gordon, Melita A. Gordon

**Affiliations:** a Department of Clinical Infection, Microbiology and Immunology, Institute of Infection and Global Health, University of Liverpool, UK; b Department of Clinical Sciences, Liverpool School of Tropical Medicine, UK; c Malawi-Liverpool-Wellcome Trust Clinical Research Programme, College of Medicine, Queen Elizabeth Central Hospital, Malawi

**Keywords:** Salmonella, Ty21a, T cells, Cytokines, Immunoglobulins

## Abstract

**Background:**

We have previously demonstrated that polyfunctional Ty21a-responsive CD4^+^ and CD8^+^ T cells are generated at the duodenal mucosa 18 days following vaccination with live-attenuated *S*. Typhi (Ty21a). The longevity of cellular responses has been assessed in peripheral blood, but persistence of duodenal responses is unknown.

**Methods:**

We vaccinated eight healthy adults with Ty21a. Peripheral blood and duodenal samples were acquired after a median of 1.5 years (ranging from 1.1 to 3.7 years) following vaccination. Cellular responses were assessed in peripheral blood and at the duodenal mucosa by flow cytometry. Levels of IgG and IgA were also assessed in peripheral blood by enzyme-linked immunosorbent assay.

**Results:**

No T-cell responses were observed at the duodenal mucosa, but CD4^+^ T-cell responses to Ty21a and FliC were observed in peripheral blood. Peripheral anti-lipopolysaccharide IgG and IgA responses were also observed. Early immunoglobulin responses were not associated with the persistence of long-term cellular immune responses.

**Conclusions:**

Early T-cell responses which we have previously observed at the duodenal mucosa 18 days following oral vaccination with Ty21a could not be detected at a median of 1.5 years. Peripheral responses were observed at this time. Immunoglobulin responses observed shortly after vaccination were not associated with cellular immune responses at 1.5 years, suggesting that the persistence of cellular immunity is not associated with the strength of the initial humoral response to vaccination.

## 1 Introduction

*Salmonella enterica* serovar Typhi (*S*. Typhi) is a facultative intracellular pathogen and the causative agent of typhoid fever. This bacterium, which is restricted to its human host, is spread via the faecal-oral route, and causes systemic illness following invasion via the mucosal surface of the small intestine [1]. A live-attenuated oral vaccine, designated Ty21a, was developed in the 1970s [2]. Vaccination with three doses of Ty21a is moderately protective, with a calculated cumulative efficacy of 48% between two and half and three years following vaccination [3]. It is estimated that 58% of all cases of disease in endemic regions occur in children under 5 years [4]. Although Ty21a has not been routinely administered in children, it has been demonstrated that when administered in liquid suspension, Ty21a is immunogenic in children aged between 2 and 6 years [5], [6].

Ty21a is able to induce humoral and cellular immune responses, both of which have been implicated in protection against disease [7]. The peripheral humoral response to Ty21a has not previously been assessed beyond 42 days [8]; however, one novel live-attenuated oral vaccine candidate, CVD 909, has demonstrated the capacity to generate memory B cells which persist for at least one year [9]. Peripheral cellular responses targeting soluble *S.* Typhi flagella (FliC) as well as infected host cells have been assessed following vaccination with Ty21a, and data indicate that T cells responding to these antigens can persist for at least two years post-vaccination [10], [11], [12].

Recently, controlled human infection has demonstrated that polyfunctional CD8^+^ T cells are associated with protection against disease when volunteers are challenged with approximately 10^3^ CFU [13], but are associated with an increased susceptibility to disease when volunteers are challenged with approximately 10^4^ CFU [14]. It has been suggested that higher dose inoculum generates stronger inflammatory responses than the lower dose inoculum and that exposure to this inflammatory environment may favour systemic dissemination [14]. Thus, polyfunctional CD8^+^ T cells do appear to play a dominant role in protection against typhoid fever in humans. We have previously demonstrated that vaccination with Ty21a generates robust, polyfunctional CD4^+^ and CD8^+^ T-cell responses at the duodenal mucosa and in peripheral blood at day 18 [7]; however, whether early duodenal responses persist in the long-term has yet to be determined.

An increased understanding of the longevity of immune responses both at the intestinal mucosa and in peripheral blood may allow us to identify both early and late functional correlates of vaccine-mediated protection, which are currently unknown. Here, we have assessed humoral immunity in peripheral blood and cellular immunity at the duodenal mucosa and in peripheral blood approximately 1.5 years following oral vaccination with Ty21a, and compared responses with those observed in a control group. These data provide a unique insight into the longevity of human mucosal and peripheral immune defence.

## 2 Materials and methods

### 2.1 Ethical approval, recruitment, and study protocol

All volunteers provided written informed consent. This study was approved by the United Kingdom National Research Ethics Service (13/NW/0282). Eighteen healthy adult volunteers were enrolled into the study. Ten volunteers (5 males and 5 females; median age 24 years) were recruited to an unvaccinated control group. Eight volunteers (3 males and 5 females; median age 23.5 years) who had previously been vaccinated with live-attenuated *S*. Typhi (Ty21a; Vivotif®) as part of a previous study (10/H1005/20) were recalled (Table 1). During the previous study, volunteers were vaccinated with live-attenuated *S*. Typhi (Ty21a; Vivotif®), according to the manufacturer’s instructions – a single oral capsule was taken on days 0, 2 and 4, approximately 1 h before a meal with a cold or lukewarm drink. Since we wished to assess the longevity of responses generated from the original vaccination, recalled volunteers were not revaccinated. Full details of the previous study (10/H1005/20) are presented in the Supplementary Material and Methods .

**Supplementary material:** 

### 2.2 Mucosal mononuclear cell (MMC) isolation

Mucosal samples were acquired approximately 1.5 years following vaccination. O_2_ was administered nasally, and saturation was monitored throughout endoscopic biopsy. Sedation was offered to all volunteers; those who re- quested sedation were given up to 5 mg of midazolam intravenously. By use of large-capacity forceps (Boston Scientific), 12–15 single-bite mucosal biopsy specimens were acquired during flexible video-endoscopy from the duodenal mucosa at parts D2-D3 (n = 16) MMCs were isolated from biopsy specimens, using a modified version of a previously described method [15]. Full details are presented in the Supplementary Materials and Methods.

### 2.3 Peripheral blood mononuclear cell (PBMC) isolation

Peripheral blood samples were collected in lithium heparin Vacutainers (BD Biosciences) (n = 17) 6 days prior to mucosal sampling. PBMCs were isolated using Histopaque-1077™ (Sigma-Aldrich), according to the manufacturer’s instructions. Full details are presented in the Supplementary Materials and Methods.

### 2.4 Antigenic stimulation and incubation

PBMCs (1 × 10^6^ cells/well) and MMCs (approximately 1 × 10^6^ cells/well) were seeded in complete medium in 96 well v-bottom plates. Cells in each well were stimulated with either 5 × 10^6^ colony forming units (CFU) heat-killed *Salmonella* Typhi Ty21a (Vivotif; suspended in Dulbecco’s PBS, quantified using the Miles and Misra technique, and killed by incubation at 95 °C for 30 min) or 10 ng FliC protein flagella. One positive control well was stimulated with 100 ng staphylococcal enterotoxin B (SEB; Sigma-Aldrich). One negative control well was left untreated to adjust for non-antigen-specific background cytokine production. Cells were then incubated at 37 °C in 5% CO_2_. After 2 h, 1 µL brefeldin A (BD GolgiPlug; BD Biosciences) and 1 µL monensin (BD GolgiStop; BD Biosciences) was added to each well, and the plate incubated for a further 16 h at 37 °C in 5% CO_2_.

### 2.5 Flow cytometric analyses

Following incubation, PBMCs and MMCs were washed, stained for viability and surface phenotype and, following fixation and permeabilisation, stained for intracellular cytokine production. Details of the antibodies that were used are presented in the Supplementary Materials and Methods. Cells were washed, resuspended and stored in the absence of light at 4 °C until data were acquired using a LSR II flow cytometer (BD Biosciences). Compensation beads (BD Biosciences) were used to create compensation matrices and sequential cell isolation used to identify populations of interest (Fig. 2). Full details are presented in the Supplementary Materials and Methods.

### 2.6 Enzyme-linked immunosorbent assay (ELISA)

Each well in flat-bottomed 96-well microtitre plates (Nunc) was coated with 100 µL carbonate-bicarbonate buffer containing either 50 ng *S*. Typhi lipopolysaccharide (LPS; Sigma-Aldrich) and incubated at 4 °C overnight. Plates were washed 3 times with PBS-Tween. Plates were blocked with 1.0% bovine serum albumin and incubated for 2 h at room temperature. A standard was created using serum obtained from a convalescent patient with a diagnosis of typhoid. Volunteer samples were diluted 4 times across an optimised range for optimum comparison against the standard. Plates were washed, samples were added in duplicate and incubated at 4 °C overnight. For detection of immunoglobulin G (IgG), plates were washed and incubated with 1:4000 anti-human IgG-alkaline phosphatase (Sigma-Aldrich) for 2 h. For detection of immunoglobulin A (IgA), plates were washed and incubated with 1:4000 anti-human-IgA (AbD Serotec) for two hours; plates were washed again and then incubated with 1:2000 streptavidin to alkaline phosphotase (AbD Serotec) for 1 h. For detection of both IgG and IgA, plates were washed and incubated with 100 µL *p*-nitrophenyl phosphate (Sigma-Aldrich). Optical density was measured at 405 nm using a FLUOstar Omega ELISA plate reader (BMG Labtech).

### 2.7 Statistical analyses

Comparisons were made using paired and unpaired *t* tests based on 1000 bootstrapped samples, as indicated. Statistical analyses were performed using SPSS v22 (IBM). Differences were considered significantly different if bootstrapped confidence intervals did not cross zero.

## 3 Results

### 3.1 Recruitment and sampling

Eight volunteers who had previously been vaccinated as part of past studies were recalled for sampling. The period between vaccination and sampling varied, with the median period between vaccination and sampling at 1.5 years (Table 1; range from 1.1 to 3.7 years).

**Table 1:** Volunteer demographics, vaccination and sampling information.

Study group	Study number	Gender	Age	Time from vaccination to sampling	
				Years	Median (Years)
Vaccine	4539/02	F	20	1.6	1.5
	4539/03	F	18	1.4	
	4539/05	F	30	1.4	
	4539/07	F	20	1.1	
	4539/09	F	26	1.5	
	4539/12	M	21	1.6	
	4539/13	M	22	1.5	
	4539/14	M	37	3.7	

Control	4539/01	F	22	N/A	N/A
	4539/04	F	22		
	4539/08	F	26		
	4539/10	F	23		
	4539/11	M	24		
	4539/15	F	26		
	4539/16	M	25		
	4539/17	M	23		
	4539/18	M	35		
	4539/19	M	20		

### 3.2 Serum immunoglobulin specificity

Ty21a-mediated protection is dependent upon the expression of LPS [2]. Although humoral responses to LPS are not believed to confer protection at an individual level, in field trials, they have been shown to correlate with overall vaccine efficacy and are useful measures of immunogenicity [16], [17], [18]. We measured levels of serum anti-LPS IgG and IgA in vaccinated volunteers and controls.

**Fig. 1:**
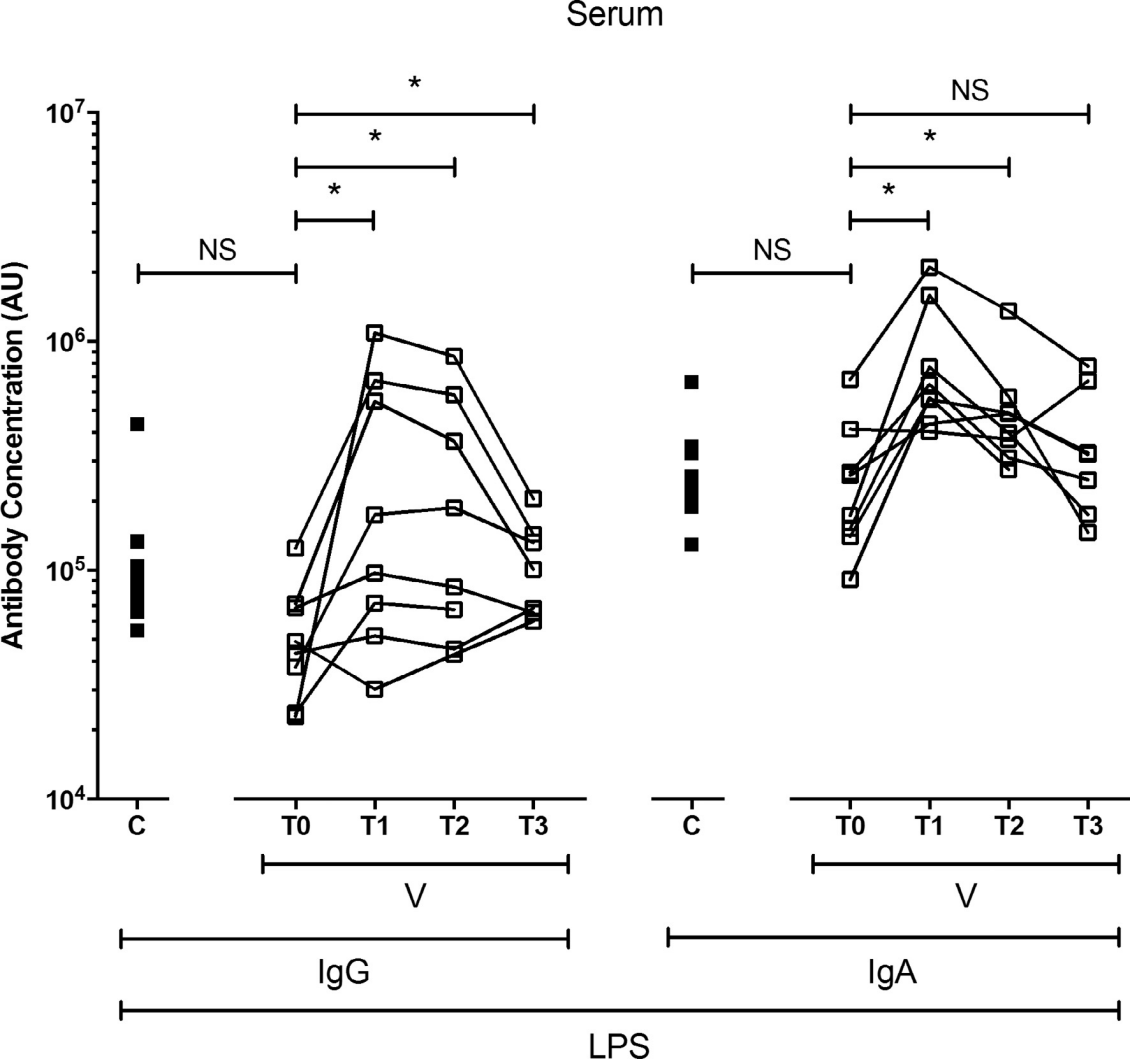
Levels of serum immunoglobulin (Ig)G and IgA to *Salmonella* Typhi lipopolysaccharide (LPS). The levels of IgG and IgA specific to *Salmonella* Typhi LPS in serum, expressed in arbitrary units (AU). Unpaired comparisons were made between control (C; closed squares) and vaccinated (V; open squares) volunteers at baseline (unpaired *t* tests were performed using logarithmically transformed data). Paired comparisons were made between baseline (T0) and day 11 (T1), day 18 (T2) and approximately 1.5 years (T3) (paired *t* tests were performed using logarithmically transformed data). Horizontal bars represent mean values. Abbreviation: ns, not significant; *, bootstrapped 95% confidence interval does not cross 0.

At day 0 (baseline), levels of anti-LPS IgG and IgA did not differ between vaccinated and unvaccinated volunteers (Fig. 1). Among vaccinated volunteers, levels of anti-LPS serum IgG were 6-fold higher at day 11 (T1), 5-fold higher at day 18 (T2) and 2-fold higher at approximately 1.5 years (T3) ([bootstrapped 95% confidence interval (CI) based on arbitrary units (AU)]: −54,874 to −7404, −44,577 to −5922 and −8911 to −317, respectively; Fig. 1) than at baseline (T0).

Similarly, among vaccinated volunteers, levels of anti-LPS serum IgA were 3-fold higher among vaccinated volunteers at day 11 and 2-fold at day 18 (−97,104 to −27,173 and −41,746 to −12,843, respectively; Fig. 1) than at baseline. Levels of anti-LPS serum IgA at approximately 1.5 years were comparable with baseline.

### 3.3 Peripheral blood and gut mucosal cellular responses

**Fig. 2:**
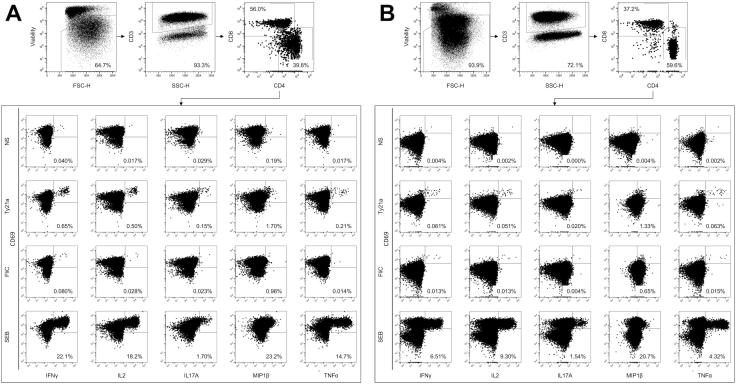
Representative flow cytometric gating strategy for intracellular cytokine analysis. Dot plots are shown for cells isolated from (A) peripheral blood and (B) the duodenal mucosa. Dead cells were removed by staining for viability (LIVE/DEAD) and gating on the negative population. T cells were identified according to the expression of CD3. T cells were classified according to the expression of CD4 and CD8 and the expression of IFN-γ, TNF-α, IL-2, IL-17A, and MIP-1β assessed in non-stimulated (NS) and in Ty21a, FliC and SEB stimulated samples. Values were expressed as the percentage of the parent CD4^+^ or CD8^+^ population.

We compared the frequency of Ty21a-responsive and FliC-responsive T cells in vaccinated volunteers and controls, at the duodenal mucosa and in peripheral blood. A combinatorial gating strategy was used to identify antigen-responsive cell populations; these were defined as the proportion of CD4^+^ and CD8^+^ T cells positive for any combination of IFN-γ ± TNF-α ± IL-2 ± IL-17A ± MIP-1β following re-stimulation (Fig. 2). Cytokine production in non-stimulated samples (negative control) was minimal, did not differ between vaccinated and unvaccinated volunteers and was subsequently subtracted from other conditions. Cytokine production in SEB-stimulated samples (positive control) was high and did not differ between vaccinated and unvaccinated volunteers.

**Fig. 3:**
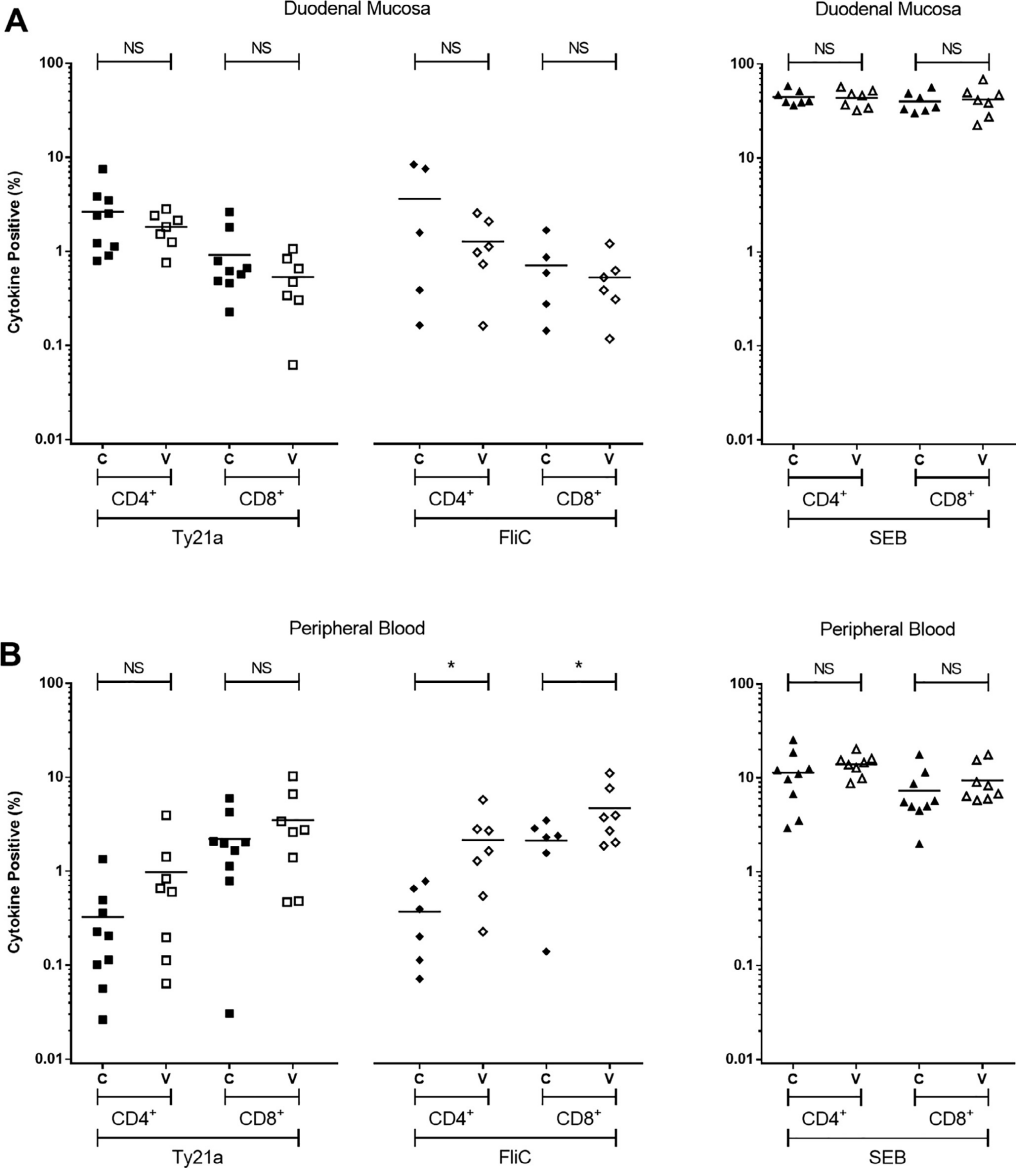
Antigen-specific cytokine-producing populations at 1.5 years. The frequency of CD4^+^ and CD8^+^*Salmonella* Typhi strain Ty21a-responsive and FliC-responsive populations expressing any combination of IFN-γ ± TNF-α ± IL-2 ± IL-17A ± MIP-1β above background. SEB-stimulated control data are also included. For control (C; closed squares, diamonds and triangles) and vaccinated (V; open squares, diamonds and triangles) volunteers, measurements were made at the duodenal mucosa (A), and in peripheral blood (B). Values are expressed as the percentage of the parent CD4^+^ or CD8^+^ population. Horizontal bars represent mean values. Abbreviation: ns, not significant; *, bootstrapped 95% confidence interval does not cross 0.

Approximately 1.5 years following vaccination, at the duodenal mucosa, the frequencies of Ty21a-responsive and FliC-responsive CD4^+^ and CD8^+^ T cells in the vaccinated group were not different from the unvaccinated control group (Fig. 3A).

Since overnight fasting, required prior to endoscopy, is known to influence cytokine production in peripheral blood in response to re-stimulation with bacterial antigens [19], we acquired non-fasting peripheral blood samples six days prior to gastroscopy. In peripheral blood, the frequency of Ty21a-responsive CD4^+^ and CD8^+^ T cells was not significantly higher in the vaccinated group at a median of 1.5 years, compared to the unvaccinated control group (Fig. 3B). The frequency of FliC-responsive CD4^+^ T cells was 6-fold higher and CD8^+^ T cells 2-fold higher in the vaccinated group at a median of 1.5 years, compared with the unvaccinated control group (([bootstrapped 95% CI based on percentage positive for combination of cytokine]: −3.48161 to −0.05863 and −5.46152 to −0.22464, respectively; Fig. 3B).

### 3.4 Characteristics and functionality of cellular responses

Polyfunctional T cells, defined as cells that express multiple cytokines/chemokines simultaneously, have been shown to correlate with vaccine-mediated protection against other intracellular infections [20], [21]. After comparing the proportions of antigen-responsive populations, we compared the cytokine expression profiles of vaccinated volunteers with those of unvaccinated volunteers. Specifically, we assessed the functionality of the response as well as individual cytokine/chemokine production.

**Fig. 4:**
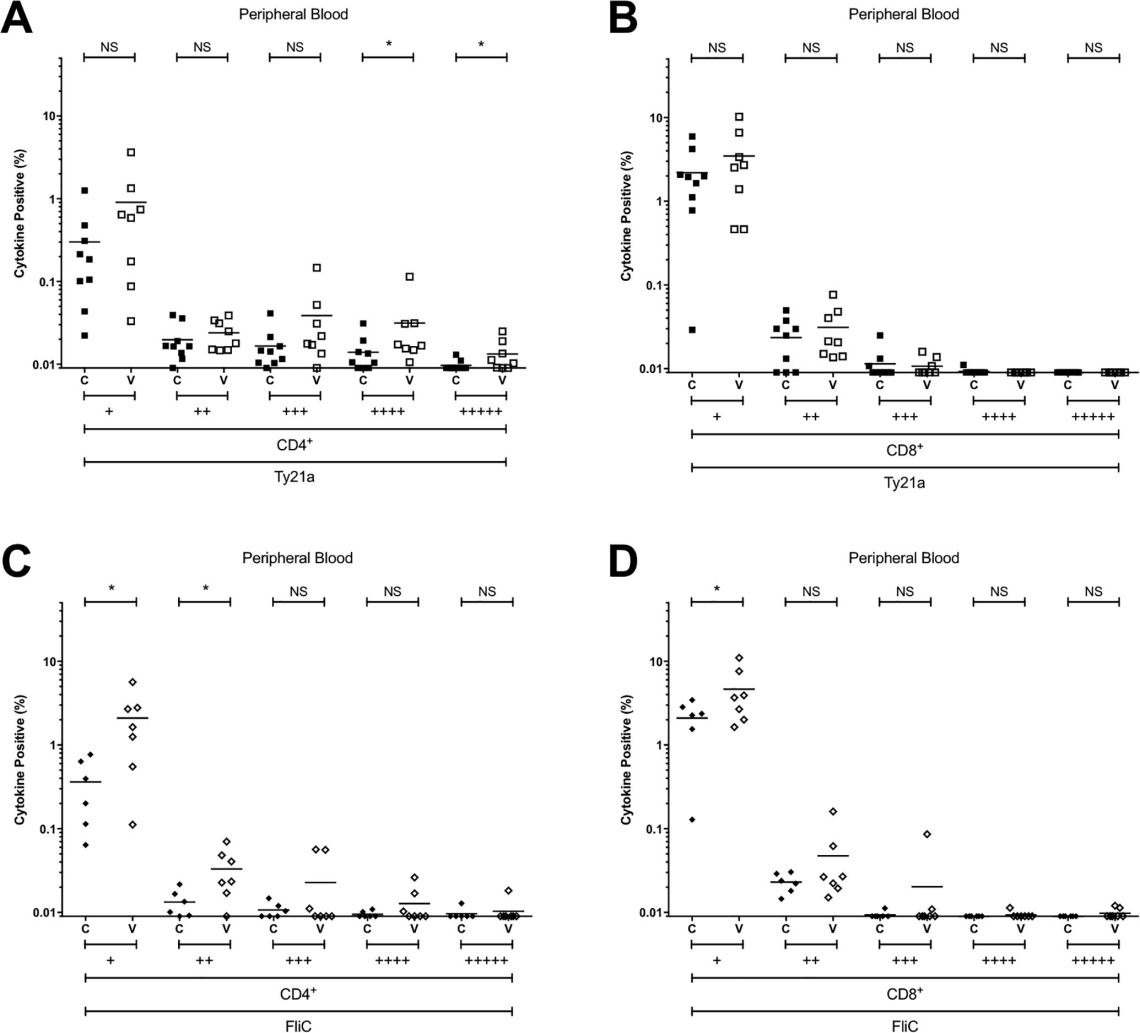
Combinations of antigen-specific cytokine production in peripheral blood. The frequency of CD4^+^ and CD8^+^ Ty21a-responsive (A and B) and FliC-responsive (C and D) populations expressing one (+), or polyfunctional populations expressing two (++), three (+++), four (++++) or five (+++++) cytokines/chemokines (IFN-γ ± TNF-α ± IL-2 ± IL1-7 ± MIP-1β) above background. For control (C; closed squares and diamonds) and vaccinated (V; open squares and diamonds) volunteers. Values are expressed as the percentage of the parent CD4^+^ or CD8^+^ population. Horizontal bars represent mean values. Abbreviation: ns, not significant; *, bootstrapped 95% confidence interval does not cross 0.

**Supplementary Fig. S1:**
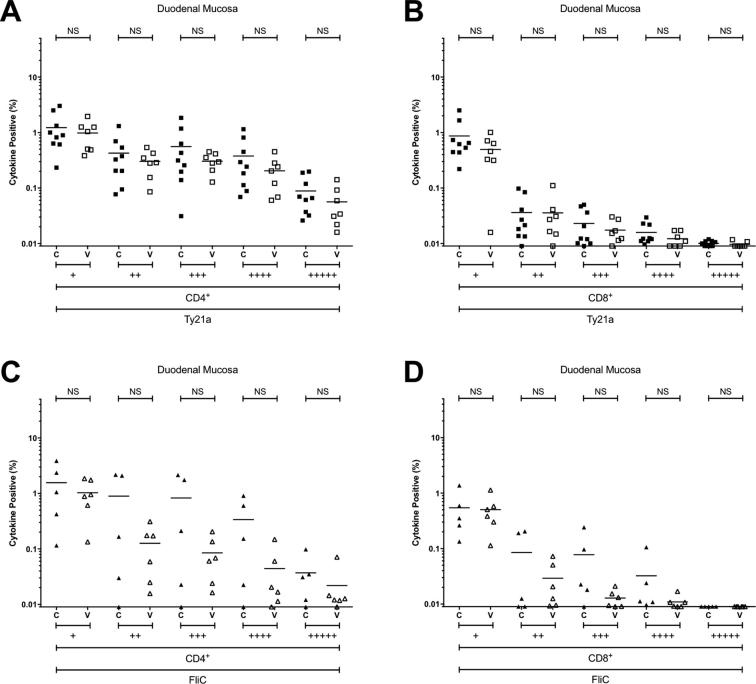
Combinations of antigen-specific cytokine production at the duodenal mucosa. The frequency of CD4+ and CD8+ Salmonella Typhi strain Ty21a-responsive (A and B) and FliC-responsive (C and D) populations expressing one (+), or polyfunctional populations expressing two (++), three (+++), four (++++) or five (+++++) cytokines/chemokines (IFN-γ ± TNF-α ± IL-2 ± IL-17A ± MIP-1β) above background. For control (C; closed squares and diamonds) and vaccinated (V; open squares and diamonds) volunteers. Values are expressed as the percentage of the parent CD4+ or CD8+ population. Horizontal bars represent mean values. Abbreviation: ns, not significant; *, bootstrapped 95% confidence interval does not cross 0.

**Supplementary Fig. S2:**
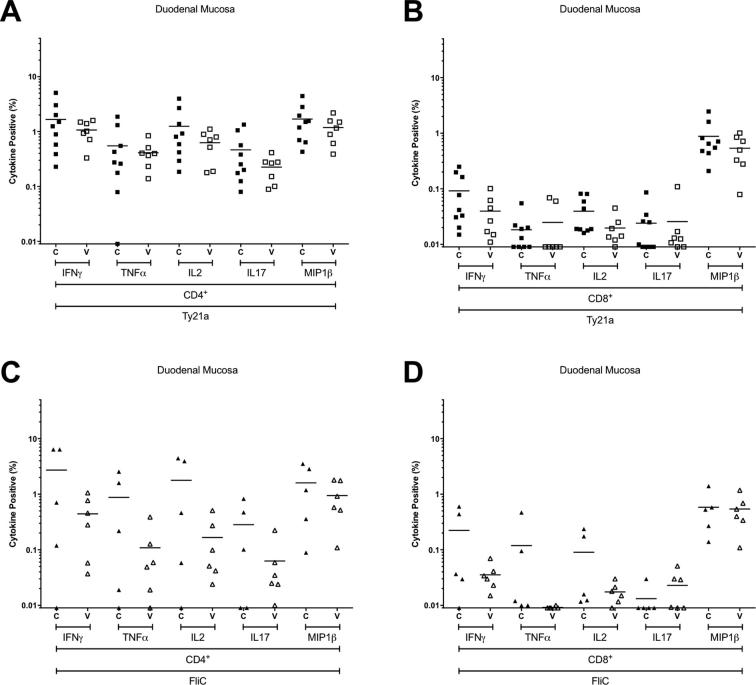
Antigen-specific production of individual cytokines at the duodenal mucosa. The frequency of CD4+ and CD8+ Ty21a-responsive (A and B) and FliC-responsive (C and D) populations expressing IFN-γ, TNF-α, IL-2, IL-17A, or MIP-1β above background. For control (C; closed squares and diamonds) and vaccinated (V; open squares and diamonds) volunteers. Values are expressed as the percentage of the parent CD4+ or CD8+ population. Horizontal bars represent mean values.

**Fig. 5:**
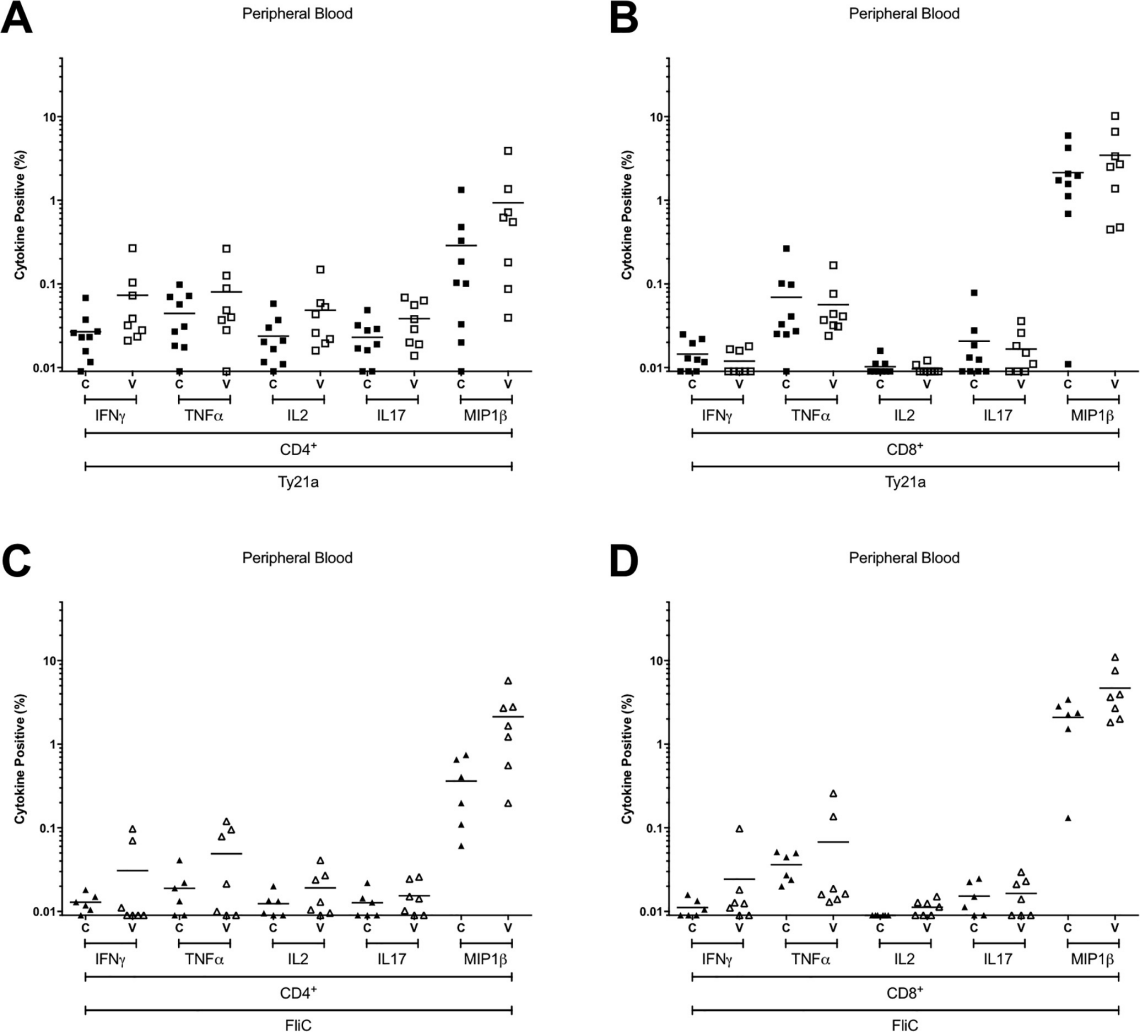
Antigen-specific production of individual cytokines in peripheral blood. The frequency of CD4^+^ and CD8^+^ Ty21a-responsive (A and B) and FliC-responsive (C and D) populations expressing IFN-γ, TNF-α, IL-2, IL-17A, or MIP-1β above background. For control (C; closed squares and diamonds) and vaccinated (V; open squares and diamonds) volunteers. Values are expressed as the percentage of the parent CD4^+^ or CD8^+^ population. Horizontal bars represent mean values.

Consistent with our published data [7], responses among CD8^+^ T cells comprised far fewer polyfunctional subpopulations, both at the duodenal mucosa and in peripheral blood (Supplementary Fig. S1 and Fig. 4). Of the cytokines/chemokines studied here, MIP1β was consistently the most commonly expressed among Ty21a-responsive and FliC-responsive CD4^+^ and CD8^+^ T-cell populations, at the duodenal mucosa and in peripheral blood (Supplementary Fig. S2 and Fig. 5).

At the duodenal mucosa, the frequencies of Ty21a-responsive or FliC-responsive CD4^+^ and CD8^+^ T cells expressing one, two, three, four or five cytokines/chemokines in the vaccinated group were not different from the unvaccinated control group (Supplementary Fig. S1). Of the cytokines studied here, no discernible difference was observed between groups at the duodenal mucosa among either CD4^+^ and CD8^+^ T cells (Supplementary Fig. S1).

In peripheral blood, the frequency of polyfunctional Ty21a-responsive CD4^+^ T cells expressing four and five cytokines/chemokines was significantly higher in vaccinated volunteers than controls (−0.04385 to −0.00047 and −0.00810 to −0.00016, respectively; Fig. 4). Consistent with the polyfunctional nature of these responses, the frequency of IFN-γ, TNF-α, IL-2, IL17 and MIP-1β expression among Ty21a-responsive CD4^+^ T cells was increased (Fig. 5). This suggests that polyfunctional Ty21a-responsive CD4^+^ T cells generated in response to vaccination persist for at least 1.5 years.

The frequency of FliC-responsive CD4^+^ T cells expressing one and two cytokines/chemokines was higher in vaccinated volunteers than controls (−3.1896 to −0.52607 and −0.03688 to −0.00417, respectively; Fig. 4). Analysis by individual cytokine revealed that the frequency of IFN-γ, TNF-α, IL-2, and MIP-1β expression among FliC-responsive CD4^+^ T cells was increased (Fig. 5).

## 4 Discussion

We have previously described the early mucosal response to live oral vaccination with Ty21a in peripheral blood and at the human duodenal mucosa; here we assessed the long-term cellular response to Ty21a at the same site and compared duodenal responses with peripheral responses. We demonstrate that, while peripheral polyfunctional cellular responses persist for at least 1.5 years, duodenal responses – which we have previously observed at day 18 [7] – do not persist. The strength of early peripheral humoral responses to *S.* Typhi LPS, which have previously been associated with protective efficacy [18], were not associated with the strength of peripheral cellular responses at approximately 1.5 years (data not shown).

In contrast with observations made at 18 days [7], no response was observed at the duodenal mucosa in either T-cell subset approximately 1.5 years following vaccination. Peripheral polyfunctional T-cell responses did persist and could be detected at 1.5 years. This suggests that local duodenal responses are more transient than peripheral responses.

For some time the generation of polyfunctional T cells has been believed to be an important factor in conferring protection against typhoid fever [12], [13], [22], [23]. Recently it has been demonstrated that, when volunteers are challenged with 10^3^ CFU, polyfunctional CD8^+^ T cells are associated with protection against typhoid fever [13]. Consistent with our previously published data [7], the frequency of responsive cells tended to be higher amongst CD4^+^ T cells; this is likely due to our use of soluble antigen preparations, which are dependent upon cross-presentation to engage cytotoxic CD8^+^ T cells [24], [25], [26]. Interestingly, while Ty21a-responsive CD4^+^ T cells comprised subsets which simultaneously expressed four and five cytokine/chemokines, FliC-responsive CD4^+^ T cells tended to express just one or two cytokines/chemokines. This suggests that the FliC antigen alone is not responsible for the generation of polyfunctional responses and that other antigens, which are present in the heat-killed Ty21a preparation, are responsible for the induction of polyfunctional responses, which are more likely to play a role in protection against disease.

The fact that duodenal responses are transient, may indicate that the composition of mucosal T-cell populations is subject to considerable change. This may be due to the relatively high frequency with which different pathogens are encountered at the intestinal mucosa, and rapid changes in the regulation of mucosal homing ligands. It has previously been demonstrated that peripheral responses, generated through vaccination with Ty21a, with increased mucosal homing potential, persist in peripheral circulation for at least 90 days [25]. Thus, if *S*. Typhi were to be re-encountered at the intestinal mucosa, long-lived peripheral cell populations would likely possess an enhanced capacity to rapidly migrate to the mucosal surface through innate signalling and homing receptor up regulation [27].

Consistent with our previously published observations [7], all volunteers had detectable baseline levels of serum immunoglobulin specific to *S.* Typhi LPS. This may be the result of immunoglobulin binding to the core, or as a result of environmental exposure to non-typhoidal strains bearing the same LPS O-antigens as *S.* Typhi (O-9 and O-12) [28]. Not all vaccinated volunteers generated peripheral humoral anti-LPS responses; likely a reflection of the known limited efficacy of this vaccine [3]. While levels of serum anti-LPS IgG were significantly higher across all 3 time points, the strength of early responses steadily declined. Similarly, early anti-LPS IgA responses steadily declined, being significantly higher at day 11 and day 18, but returning to a level which was comparable with baseline at 1.5 years. In humans, memory B cells can persist for the life of the host (more than 50 years); however, typically, levels of circulating immunoglobulin decline rapidly following clearance of the antigen [29], [30], [31]. Thus, if the antigen is not reencountered *in vivo*, immunoglobulin responses are unlikely to be detected in the long-term.

The assessment of baseline immunoglobulin levels indicates that groups were well matched for prior exposure to *S.* Typhi. We have previously observed that heterologous influenza-responsive T-cell responses are generated at the duodenal mucosa 18 days following vaccination with Ty21a. Unfortunately, we were unable to determine the longevity of these responses in this study as the majority of the vaccinated cohort had been vaccinated against influenza in the period since being vaccinated with Ty21a (data not shown). We assessed the expression of four cytokines and one chemokine, as a result, the T-cell populations identified here are unlikely to represent the responsive populations in their entirety. While we have focused on the assessment of mucosal cellular immune defence, further studies which analyse interplay between cellular and humoral immune defence at the intestinal mucosa would provide us with a more comprehensive overview of the human immune response to vaccination. The use of intestinal lavage has been used in other studies to quantify IgA in mucosal secretions [32]. Variation within the human population may mean that we are underpowered to observe some other important biological effects.

Taken together, our data demonstrate that early intestinal responses do not persist locally at the duodenal mucosa in the long-term. The fact that oral vaccination does generate peripheral populations which persist for at least 1.5 years supports the development of next-generation oral vaccines targeting typhoid, since it demonstrates longevity of orally induced cellular responses. Data presented elsewhere indicate that populations generated through oral vaccination express the homing molecules necessary to rapidly migrate to the intestinal mucosa following pathogenic exposure [25]. Controlled human infection, post-vaccination, may allow us to identify mechanisms responsible for efficacious defence against pathogens encountered at the intestinal mucosa and in the peripheral circulation.

## Conflict of interest

None.
