# Supplementary material for: Longevity of duodenal and peripheral T-cell and humoral responses to live-attenuated Salmonella Typhi strain Ty21a
Source: Vaccine. 2018 Jul 25;36(31):4725–33. doi: 10.1016/j.vaccine.2018.05.114 (PMC6041722; doi:10.1016/j.vaccine.2018.05.114)
Supplement: Supplementary file 1 [file mmc1.docx]

**SUPPLEMENTARY MATERIALS AND METHODS**

**Description of past study protocol *(10/H1005/20)***

Healthy adult volunteers were enrolled into the study and vaccinated with live-attenuated *S*. Typhi (Ty21a; Vivotif®), according to the manufacturers instructions – a single oral capsule was taken on days 0, 2 and 4, approximately 1 hour before a meal with a cold or lukewarm drink. Peripheral blood and mucosal samples were acquired at day 18 using the same methods described here.

**MMC isolation**

Biopsies were collected on ice in R15 medium (RPMI-1640 supplemented with 15% foetal bovine serum (FBS), 2% 200 mM L-glutamine and antibiotic/antimiotic). Biopsies were incubated in CII-S medium (R15 medium supplemented with collagenase II-S (Sigma-Aldrich) (0.5 mg/mL)) at 37°C for 30 minutes at a 45° angle with shaking (220 rpm). Mucosal biopsies were mechanically disrupted by passing the tissue suspension through a 16-gauge blunt-ended needle five times. The suspension was then passed through a 70 µm cell strainer and the cell suspension stored on ice. Tissue fragments captured in the cell strainer were transferred back into the original tube by rinsing the cell strainer with CII-S medium. The process of incubation, disruption and filtration was then repeated twice more. Cell suspensions were pooled, washed, counted and seeded in R20 medium (RPMI-1640 supplemented with 20% FBS, 2% 200 mM L-glutamine and antibiotic/antimiotic) in 24-well flat bottomed plates at a concentration of 1 × 10^6^ cells/well. Cells were rested overnight at 37°C in 5% CO_2_. The following day, cells were harvested, recounted, and seeded in complete medium (RPMI-1640 supplemented with 10% FBS and 2% 200 mM L-glutamine) in 96-well flat-bottomed plates at a concentration of approximately 1 × 10^6^ cells/well.

**Peripheral blood mononuclear cell (PBMC) isolation**

Peripheral blood samples were diluted with an equal volume of Dulbecco's phosphate-buffered saline (Dulbecco’s PBS) (Invitrogen) and PBMCs isolated by differential centrifugation using Lymphoprep™ (Axis-Shield). Cells were washed twice in Dulbecco’s PBS, counted and seeded in complete medium in 96-well flat-bottomed plates at a concentration of 1 × 10^6^ cells/well.

**Flow cytometric analyses**

Following incubation, PBMCs and MMCs were washed and stained for viability (Vivid®; Invitrogen) and surface phenotype using antibodies specific to CD3-BV510 (OCT3; BioLegend), CD4-PE-Cy7 (SK3; BD Biosciences), CD8-APC-H7 (SK1; BD Biosciences), CD14-HorizonV450 (MφP9; BD Biosciences) and CD19-HorizonV450 (HIB19; BD Biosciences). Following fixation and permeabilisation (Cytofix/Cytoperm™; BD Biosciences), cells were stained for intracellular CD69-PE/CF594 (B27; BD Biosciences), macrophage inflammatory protein (MIP)-1β-APC (24006; R&D Systems), interferon (IFN)-γ-AF700 (B27; BD Biosciences), tumour necrosis factor (TNF)-α-AF488 (MAb11; BD Biosciences), interleukin (IL)-2-PE (MQ1-17H12; BD Biosciences) and IL-17A-PE (eBio64DEC17; eBioscience). Cells were washed in Perm/Wash™ (BD Biosciences), resuspended in CellFIX™ (BD Biosciences), and stored in the absence of light at 4°C until data were acquired using a LSR II flow cytometer (BD Biosciences).

Compensation beads (BD Biosciences) were used to create compensation matrices and sequential cell isolation used to identify populations of interest (Figure 2). IFN-γ^+^, TNF-α^+^ and IL-2^+^ populations were positively identified and combinatorial expression profiles determined using FlowJo version 10 (Treestar Inc.). Values were expressed as the percentage of the parent CD4^+^ or CD8^+^ population.
